# EHMTI-0205. Methodology guidelines for population surveys of headache prevalence, burden and cost

**DOI:** 10.1186/1129-2377-15-S1-B36

**Published:** 2014-09-18

**Authors:** L Stovner, M Al Jumah, G Birbeck, G Gururaj, R Jensen, Z Katsarava, L Queiroz, A Scher, R Tekle-Haimanot, S Wang, T Steiner

**Affiliations:** 1 Department of Neuroscience, Norwegian University of Science and Technology, Trondheim, Norway; 2 Prince Mohammed Ben Abdulaziz Hospital, King Saud Ben Abdulaziz University for Health Sciences, Riyadh, Saudi Arabia; 3 Department of Neurology, University of Rochester, Rochester, USA; 4 Department of Neurology, National Institute of Mental Health and Neurosciences, Bangalore, India; 5 Danish Headache Centre Glostrup Hospital, University of Copenhagen, Copenhagen, Denmark; 6 Evangelical Hospital Unna, University of Duisburg-Essen, Unna, Germany; 7 Department of Neurology, Federal University of Santa Catarina, Florianopolis, Brazil; 8 Department of Preventive Medicine and Biometrics, Uniformed Services University, Bethesda, MD, USA; 9 School of Medicine Department of Neurology, Addis Ababa Addis Ababa University, Addis Ababsa, Ethiopia; 10 The Neurological Institute, Taipei Veterans General Hospital, Taipei, Taiwan

## Background

The global burden of headache is very large, but knowledge of it is far from complete. Published population-based studies have used variable methodology, which has influenced findings and made comparisons difficult.

## Aim

To produce consensus-based methodological guidelines [1] with the main focus on migraine, tension-type headache and medication-overuse headache, but not intended to be exclusive to these.

## Method

An expert consensus group including experience and competence in headache epidemiology and epidemiology in general was drawn from all six WHO world regions. Drafts were discussed and revised by email before and after a three-day consensus conference in September 2011, and eventually revised after a public consultation posting on the International Headache Society website.

## Results

The recommendations cover most methodological issues: study design; definition of population of interest; control of bias, sample selection and participation rate; how to access and engage participants, and methods of enquiry; case definition and diagnosis, and algorithm for making headache diagnoses; use of pilot studies; measurement of headache burden. There are also discussions of how to report studies and evaluate other studies, as well as of ethical issues.

## Conclusion

The principles should be useful to researchers whose main interests are in the field of headache, but they also seek to encourage collaborations between specialists in headache disorders and epidemiologists.

No conflict of interest.
